# Gastric cancer after gastric bypass with fundectomy: The possibility for early diagnosis

**DOI:** 10.1016/j.ijscr.2019.01.040

**Published:** 2019-02-01

**Authors:** Marco Antonio Zappa, Maria Paola Giusti, Elisa Galfrascoli

**Affiliations:** a Department of General Surgery, Fatebenfratelli Hospital, Piazzale Principessa Clotilde, 3, 20121, Milano, MI, Italy; b Department of General Surgery, Fatebenefratelli Hospital, Via fatebenefraatelli 20, 22036, Erba, CO, Italy

**Keywords:** Gastric bypass, Obesity, Gastric cancer, Excluded stomach, Endoscopy, Bariatric surgery

## Abstract

Introduction: Roux-an-Y gastric bypass (RYGP) is one of the most important bariatric procedures and its results are well known in terms of weight loss and comorbid improvement. The major limitation of this technique is the difficult exploration of the excluded stomach and duodenum. Some Authors are performing the gastric bypass with fundectomy and, according to Literature, it is feasible and effective, with major advantage of explorable gastric pouch.

Presentation of case: We report the case of a 54-year-old woman affected by obesity (BMI 49 kg/m^2^). After a pre-operative multidisciplinary evaluation and gastroscopy, she underwent a laparoscopic RYGB with fundectomy in October 2016.

One year after surgery she contacted the department for vomiting, pyrosis and weakness.

Thanks to the characteristics of the surgical technique it was possible to easily perform an OGD that detected an antral ulcer. The byopsy revealed a gastric adenocarcinoma.

A degastroresection was performed and the istological finding was a gastric adenocarcinoma pT1b N0 G3.

Discussion: Early diagnosis is essential in gastric tumors to ensure a good prognosis and the gold standard is performing gastroscopy with biopsies.

With the standard technique is very challenging to perform an OGD and the cancer stage is likely to be advanced at diagnosis, with a bad prognosis for the patient.

Conclusion: From the clinical case described and the analysis of the Literature, the advantages of this technique are clear, allowing for an easy endoscopic evaluation of gastric walls with the possibility of diagnosing early stage tumors with a better outcome for patients.

## Highlights

• RYGB is the most important bariatric procedure worldwide.
• The RYGB mayor limitation is the difficult exploration of the excluded stomach and duodenum.
• The gastric bypass with fundectomy allowed for an easly endoscopic evaluation.
• The possibility to easly perform OGD guaranteed the detection of gastric carcinoma at an early stage.

## 1 Introduction

This work has been reported in line with the SCARE criteria. [1]

Gastric bypass is one of the most widespread bariatric procedures in the world due to its effectiveness in terms of weight loss and improvement in comorbidities [2].

Nevertheless, the difficulty of explorating the excluded stomach area and the consequent inaccessibility of the biliary tract, represent two major disadvantages particularly considering the high incidence of tumours in obese patients and the characteristics of this specific procedure that can cause a predisposition to the formation of gallstones [[3], [4], [5]].

In the literature cases of gastric cancer have been reported in patients who underwent gastric bypass, and diagnosis of these patients was difficult due to the drawbacks described above, with consequent diagnostic delay and poor prognostic outcomes [[6], [7], [8], [9], [10]].

**Fig. 1:**
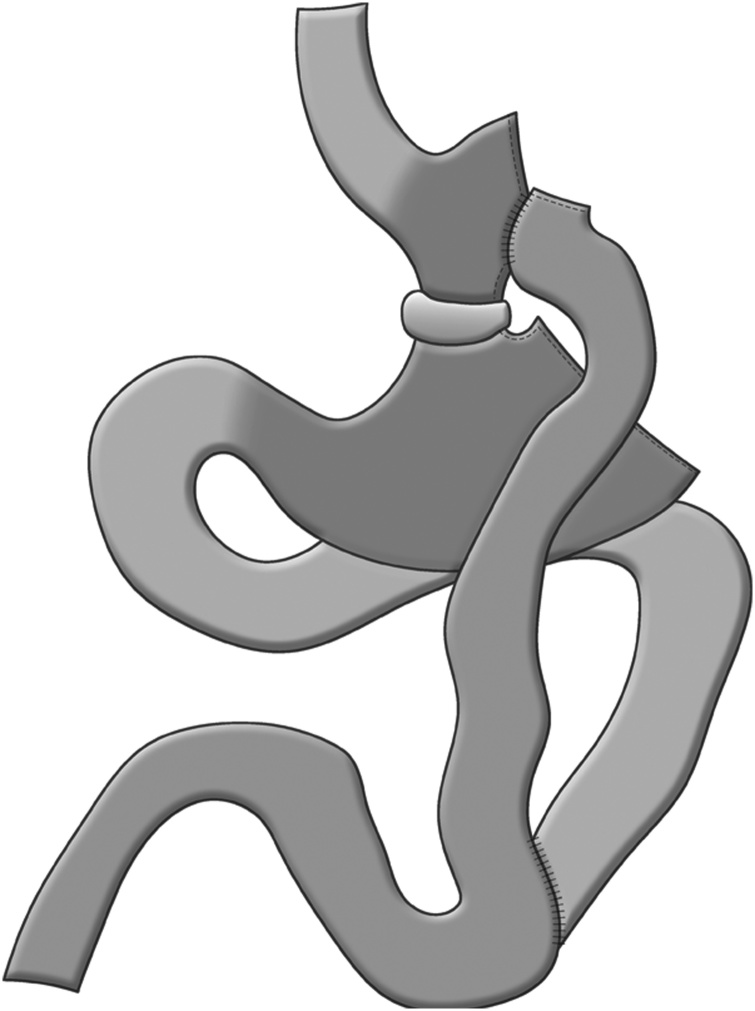
The model of gastric bypass with fundectomy and exploration of the stomach.

Consequently for some years, the authors have been using a gastric bypass technique with fundectomy and stomach exploration that, in addition to offering similar results to the standard technique, allows access to the bypassed stomach by positioning a e-PTFE band below the gastro-jejunal anastomosis that is calibrated on a 36 Fr bougie so as to enable the passage under pressure of an endoscope but to exclude the gastric antrum from the passage of the food bolus (Fig. 1).

The results in terms of outcomes and complications are comparable to those of the standard technique but with the great advantage that endoscopic retrograde cholangiopancreatography (ERCP) and biliary drainage are easily performed, as is diagnostic gastroscopy, as shown in the case presented [11,12].

## 2 Case presentation

In this article, we present the case of a 54-year-old woman suffering from severe obesity with a body mass index (BMI) of 47.5 kg / m2 (weight: 104 kg, height 148 m). In anamnesis, chronic bronchopathy treated with corticosteroids and laparoscopic cholecystectomy, no family history of cancer.

She contacted our institute for bariatric surgery. The cross-disciplinary pre-operative assessment included clinical psychology interview, dietary evaluation, oesophagogastroduodenoscopy with biopsy, *H. pylori* identification and abdominal ultrasound. All the tests were negative for pathologies and, in agreement with the patient and the cross-disciplinary team, a surgical recommendation was given. A gastric bypass with fundectomy was performed laparoscopically.

At one and three postoperative months the patient showed a good weight loss: 95 kg at one month with 18.3% excess weight loss (%EWL) and 8.7% total body weight loss; 84 kg at 3 months with a 40.6% EWL and 19.2% total body weight loss.

At nine months she had an excellent weight loss: 72 kg with a 64.9%EWL and 30.8% total body weight loss, but she reported sporadic episodes of vomiting, asthenia and poor appetite.

The latter symptoms are common after bariatric procedures and are compatible with the normal outcome of the bypass procedure. For this reason, the patient was scheduled for the normal follow-up at one year.

**Fig. 2:**
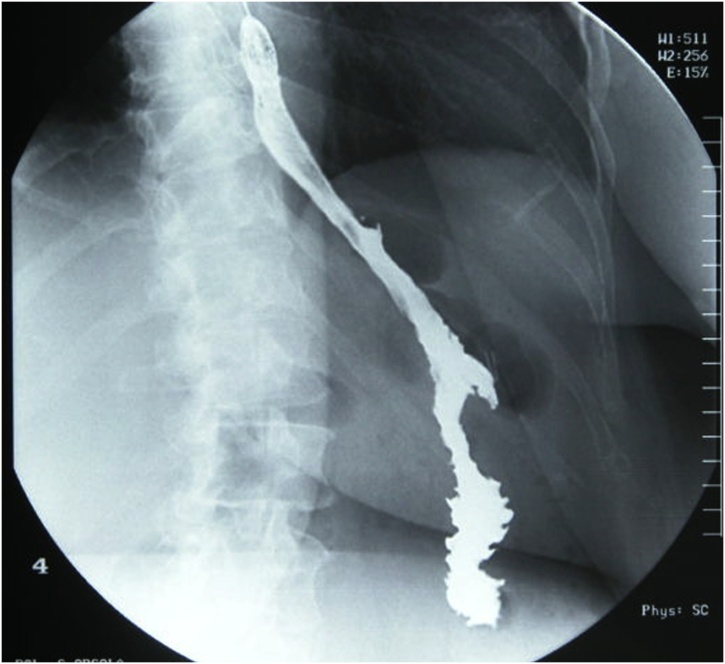
X-ray with Gastrografin demonstrating the normal progression of dye without pathological findings.

One year after surgery, the patient contacted the department because of a worsening of her symptoms with vomiting and asthenia. Blood tests and X-ray with Gastrografin were carried out. Blood tests revealed slight anaemia (haemoglobin: 10.5 g/dl), and the X-ray with Gastrografin showed a regular progression of the contrast medium through the gastro-jejunal anastomosis and an absence of pathological findings (Fig. 2).

In the context of initial uncertainty, it was possible to subject the patient to a full gastroscopy, thanks to this technique which allows for an easy exploration of the bypassed stomach. It showed the presence of a 6 mm antral ulcer on which biopsies were performed. The results indicated that it was an intestinal type G3 gastric adenocarcinoma. After this diagnosis, preoperative staging was performed with abdominal and thoracic CT scan with contrast dye, which resulted in negative metastasis and the gastric lesion was not visible. Cancer markers tested were in the normal range (CEA, Ca19.9 and alphafetoprotein).

The patient underwent laparoscopic degastroresection with D2 lymphadenectomy. The operation lasted 230 min, with no intraoperative complications. The post-operative period was normal and the patient was discharged on the fifth day.

The definitive histological analysis documented the presence of a gastric adenocarcinoma of the antrum pT1bN0M0 with 22 negative lymph nodes removed. After an oncological evaluation, the patient underwent standard follow-up without chemotherapy or radiotherapy.

The follow-up at two years showed no recurrence of disease.

## 3 Discussion

Considering the exponential increase in obesity, it has been estimated that more young patients will undergo bariatric and metabolic surgery [13]. With increasing number of bariatric procedures, surgeons all over the world have shared concerns about pathologies in the excluded gastric remnant.

The two principle disadvantages of the traditional bypass, i.e., the difficulty of explorating the bypassed stomach and the inaccessibility of the bile duct by means of classical endoscopy, have led the authors to develop a gastric bypass model that retains all the benefits of the surgery but avoids these drawbacks [11,12,14,15].

Complications in the excluded stomach after gastric bypass are infrequent but may be serious and require urgent diagnosis when they occur [16,17]. In addition to the incidence of gastric cancer, these complications include gastritis with intestinal metaplasia, haemorrhages and "bypass obstruction" syndrome (spectrum of clinical manifestations resulting from gastrectasis, which can lead to the perforation of the excluded stomach) [18,19].

Early diagnosis is essential in gastric tumours to ensure a good prognosis and the gold standard is performing gastroscopy with biopsies.

In the literature, cases of gastric adenocarcinoma and isolated cases of gastric lymphoma were reported in patients who underwent traditional bypass [3,4]. In all patients, there was a considerable diagnostic delay due to the difficulties of exploring the excluded stomach, thus resulting in advanced tumor stages at diagnosis (stage III,IV), leaving limited surgical options [[7], [8], [9], [10]].

It should also be noted that in patients undergoing gastric bypass it is difficult to identify the presence of pathologies early because symptoms are similar to those of the normal post-bariatric period such as weight loss, asthenia and possible slight anaemization.

For this reason, several authors have tried alternative methods to study the excluded portion of the stomach (double balloon endoscopy, percutaneous endoscopy, virtual endoscopy, laparoscopic gastrostomy), but each is technically challenging, often unsuccessfull or requires the patient to undergo surgery and is therefore performed only in case of necessity due to the onset of complications or when there is a high probability of malignancy. In any case, these procedures are never carried out at an early point in time [[20], [21], [22], [23]].

Biopsy findings of the main gastric chamber obtained from 40 patients undergoing double balloon endoscopy showed cases of erythematous, erosive, haemorrhagic and atrophic gastritis in 74% of patients and only 25.7% of them had normal findings [17].

The decision to modify the gastric bypass technique, backed up by extensive data in part already published and available to the authors, could represent an innovative technique to diagnose any pathology of the stomach early, and therefore recommend to the patient the ideal treatment for the best possible prognosis.

## 4 Conclusion

This article has highlighted a potentially serious situation that is not unknown to the surgeons.

The authors address the problem of early diagnosis of gastric cancer in patients who undergo bariatric surgery by proposing a laparoscopic gastric bypass model with fundectomy and explorable stomach pointing out the importance of access to the distal stomach while preserving the benefits of a weight loss procedure.

From the clinical case described and an analysis of the literature, the advantages of this technique are clear, allowing for an easy endoscopic evaluation of gastric walls with the possibility of diagnosing early-stage tumours with a better outcome for patients.

## Conflict of interest

The authors declare that they have no conflict of interest.

## Fundings

The paper has no funding.

## Ethical approval

Approved from the local ethical committee. Final judgement: approved.

## Consent

The consent to publish has been obtained from the patient and a copy of it is available for review on request.

## Author contribution

Marco Antonio Zappa, M.D. Project administrator, writing original draft.

Maria Paola Giusti, M.D. Data curation.

Corresponding author: Elisa Galfrascoli, M.D. Writing, review and editing.

## Research Registration Number

NA.

## Guarantor

Marco Antonio Zappa.

## Provenance and peer review

Not commissioned, externally peer-reviewed
